# Emergency Neurosurgery in a Patient With Pacemaker: The Double Trouble

**DOI:** 10.7759/cureus.58256

**Published:** 2024-04-14

**Authors:** Sidra Rahman, Chandini Kukanti, Niraj Kumar

**Affiliations:** 1 Neuroanesthesiology and Critical Care, All India Institute of Medical Sciences, New Delhi, New Delhi, IND

**Keywords:** intracerebral hemorrhage, emergency neurosurgery, coronary artery disease, dual-chamber pacemaker, primary decompressive craniectomy

## Abstract

Perioperative management of a patient with multiple comorbidities, being taken up for an emergency neurosurgical procedure presents a unique set of challenges to the anesthetist as it requires quick preoperative evaluation in order to avoid any delay in the surgery and limit the extent of cerebral injury. This case report highlights the perioperative management of a 55-year-old obese male patient, with a history of hypertension and coronary artery disease with a permanent pacemaker presenting to the emergency with weakness of right upper and lower limbs, suggestive of an acute stroke due to intracerebral hemorrhage. The patient was taken up for emergency decompressive craniectomy in view of increasing intracranial pressure and deteriorating consciousness. The pacemaker could not be changed to asynchronous mode in the preoperative period due to the non-availability of a magnet and trained personnel from the company of the pacemaker to change the settings immediately. Intraoperatively, all the necessary precautions for the prevention of pacemaker-related complications were followed. After the completion of the surgery, the patient was shifted to the neuro-intensive care unit for postoperative management.

## Introduction

Emergency neurosurgery in a patient with multiple comorbidities presents a challenging situation to the anesthetist as it requires quick preoperative evaluation and prompt initiation of surgery to limit the extent of cerebral injury. Emergency neurosurgical procedures are done for indications such as drainage of cerebrospinal fluid, control of intracranial pressure, and decompression of the intracranial components [1]. In patients with multiple systemic comorbidities, there is a decrease in physiological reserve due to chronic interaction of such comorbidities leading to increased risk of perioperative complications. Preoperative risk stratification and assessment of the severity of comorbidities can aid in tailoring the anesthetic plan to provide the best outcomes [2].

Hypertension is one of the leading risk factors for the development of spontaneous intracerebral hemorrhage leading to increased intracranial pressure. Uncontrolled hypertension could be due to various reasons and a common preventable reason is poor patient compliance with the anti-hypertensive regimen. Hypertension-associated hematomas are seen in the basal ganglia, pons, posterior fossa, and thalamus [3]. Hypertension, when uncontrolled, is a strong independent risk factor for the development of coronary artery disease [4]. Hence a patient with chronic uncontrolled hypertension with co-existing coronary artery disease can present to the emergency department with acute stroke due to intracerebral hemorrhage. The anesthetic goals in such patients include maintaining hemodynamic stability, avoiding acute fluctuations in blood pressure, maintaining cerebral perfusion pressure, managing raised intracranial pressure, and preventing secondary insults due to various causes such as hypoxia, hypercarbia, and hypoglycemia [5].

We describe the perioperative management of a 55-year-old obese male patient with a body mass index (BMI) of 37 kg/m^2^, uncontrolled hypertension, and coronary artery disease with a permanent pacemaker for 10 years, presenting to the emergency with acute stroke due to intracerebral hemorrhage. The intraoperative management was challenged due to the presence of a pacemaker in situ and the non-availability of trained personnel for prompt settings of the pacemaker before taking up the patient for emergency decompressive craniectomy.

## Case presentation

**Figure 1:**
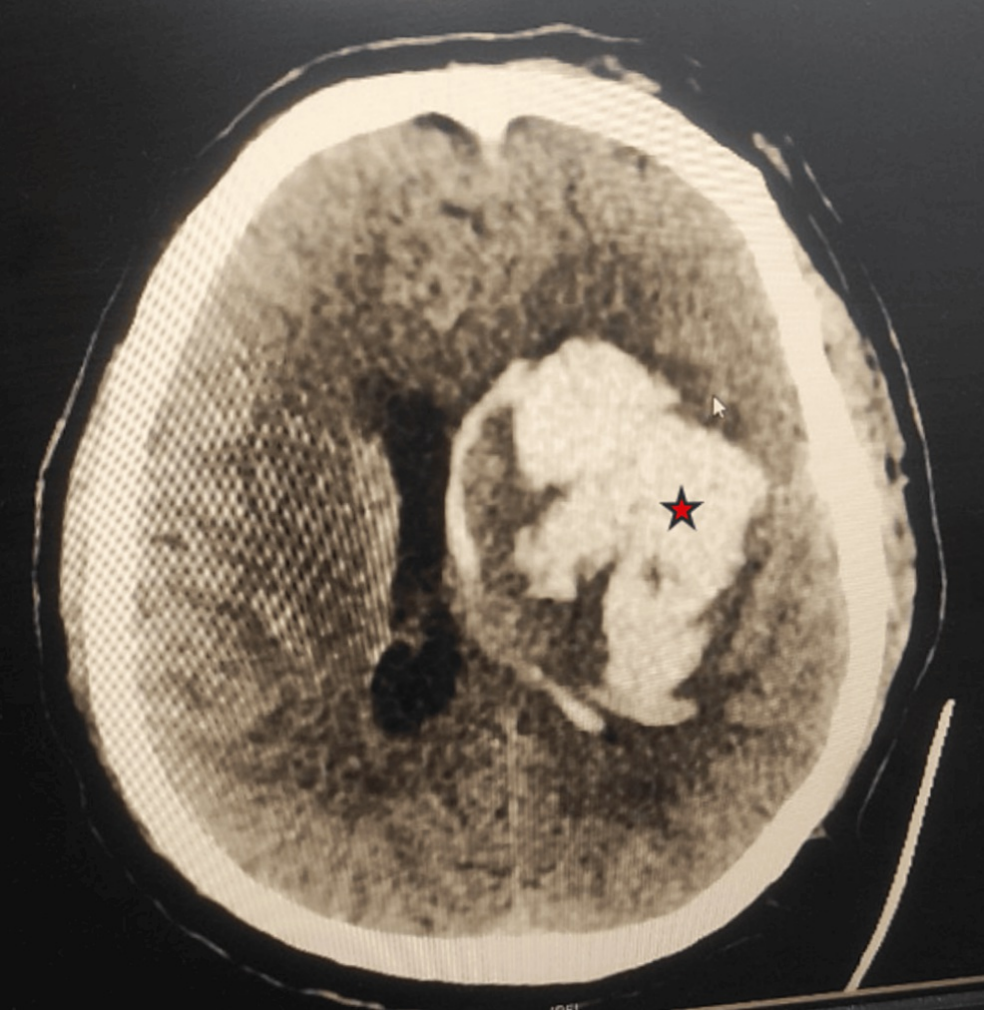
Axial section of the NCCT head at the level of the lateral ventricle showing left gangliocapsular bleed (Red Star) with intraventricular extension with mass effect and midline shift NCCT: Non-contrast computerized tomography

A 55-year-old male, known hypertensive with a history of coronary artery disease status post percutaneous transluminal coronary angioplasty (PTCA) with a permanent pacemaker (St. Jude Medical, Saint Paul, United States) insertion done 10 years ago, came with the chief complaints of weakness in the right upper and lower limbs associated with a decrease in consciousness over the last 12 hours. The patient was taken to a nearby hospital where a non-contrast computerized tomography (NCCT) head was done which showed intraparenchymal bleed involving the left basal ganglia extending superiorly to the left parietal lobe with mild adjacent perilesional edema. On arrival at our institute, the patient had chief complaints of weakness in the right upper and lower limbs associated with slurring of speech and the Glasgow Coma Scale (GCS) of the patient was E3V2M5. There was a progressive deterioration in consciousness, which raised the suspicion of an expanding hematoma. Hence a repeat NCCT head was done which showed left basal ganglia bleed with a volume of 56 ml and midline shift of 6 mm with intraventricular extension with an intracerebral hemorrhage score of 3 (Figure 1). Hence the patient was planned to be taken up for emergency left fronto-parieto-temporal decompressive craniectomy and evacuation of basal ganglia bleed.

A quick preoperative assessment was done and the anesthetic concerns were noted. The patient was a known hypertensive for 10 years on oral medications Telmisartan 40 mg once a day and Amlodipine 5 mg twice a day. However, the patient had poor compliance with the drug regimen. The vital parameters at the time of presentation were a pulse rate of 98 beats per minute, Blood pressure of 160/110 mm Hg, and saturation of 95% on room air. On assessing and obtaining the history from the patient's relative, the weight of the patient was 120 kg with a height of 180 cm and a BMI of 37 kg/m^2^. It was an anticipated difficult airway because of the heavy jaw and thick neck of the patient. On review of previous documents, the patient had a history of PTCA with pacemaker insertion done 10 years back with the pacemaker set in DDD (dual-chamber paced, dual-chamber sensed, dual response to sensing) mode and the patient was on dual antiplatelet therapy (Aspirin and Clopidogrel) which was taken even on the morning of the incident. Echocardiography reports documented at a previous hospital revealed dyskinetic right coronary artery territory, dilated left atrium, mild concentric left ventricular hypertrophy with left ventricular ejection fraction of 41%, and grade 1 diastolic dysfunction of the left ventricle.

**Figure 2:**
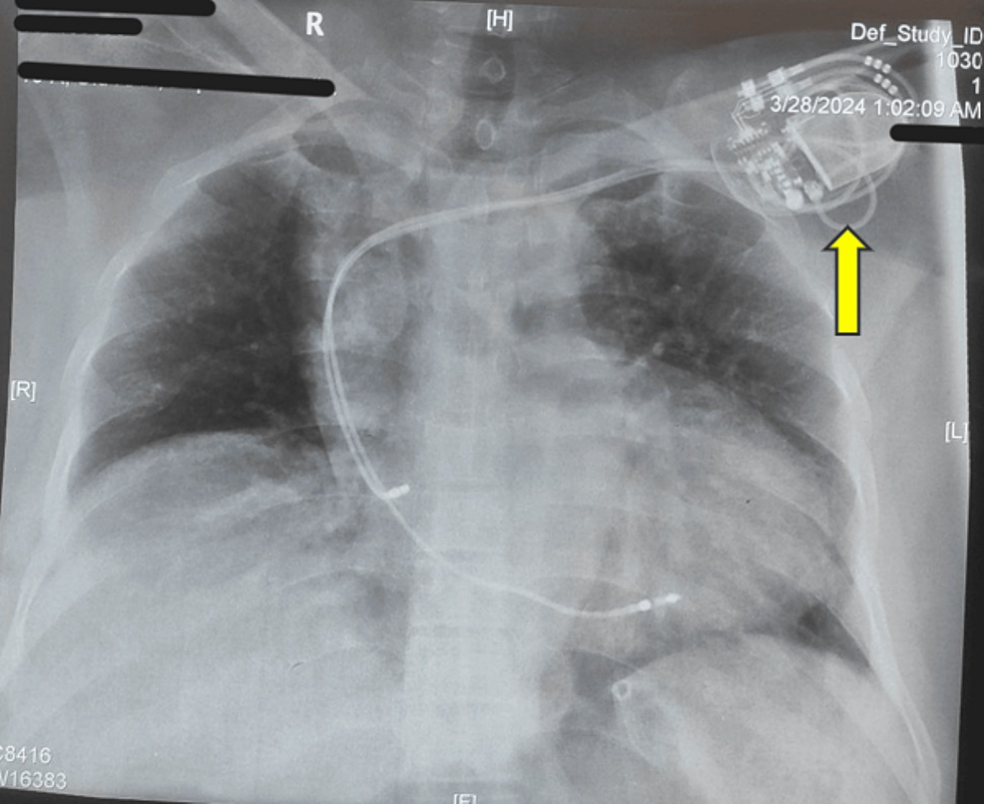
Chest X-ray of the patient showing cardiomegaly and pacemaker in situ in the left pectoral region (yellow arrow)

**Figure 3:**
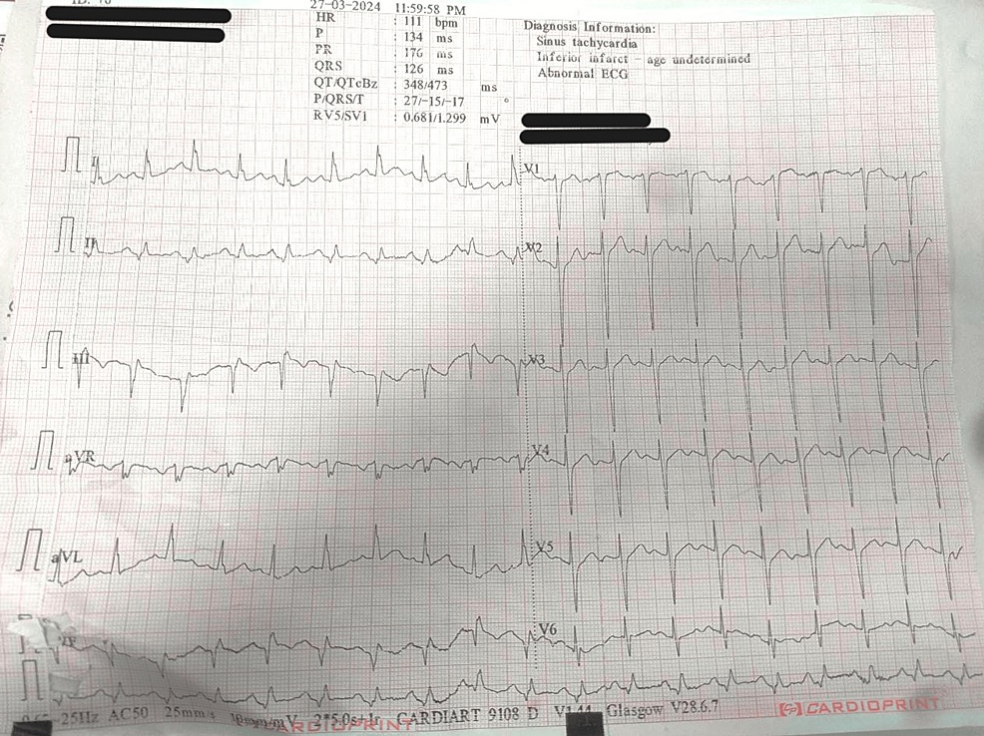
Preoperative electrocardiogram of the patient

Chest X-ray showed cardiomegaly with intact leads of the pacemaker and the lung fields were normal (Figure 2). ECG revealed sinus tachycardia with ischemic changes in the inferior wall leads (II, III, and aVF) (Figure 3). Since it was an emergency procedure and the patient's level of consciousness was deteriorating, the pacemaker could not be changed to asynchronous mode due to the non-availability of a magnet and trained personnel from the company of the pacemaker to change the settings immediately.

The patient was promptly shifted to the operation theatre. ASA standard monitors were attached. The patient was placed in a ramp position before intubation. General anesthesia was induced with intravenous Fentanyl 150 mcg, Etomidate 14 mg, and Succinylcholine 125 mg. Intubation was done with a video laryngoscope and an 8.5 polyvinyl chloride (PVC) cuffed endotracheal tube was secured in place. Invasive arterial blood pressure monitoring was done with an arterial cannula in the right posterior tibial artery. Right internal jugular vein cannulation was done under ultrasound guidance and central venous pressure monitoring was done. Anesthesia was maintained with air at 50% and oxygen at 50%, Sevoflurane, fentanyl, and rocuronium infusion. Intraoperative arrhythmias were anticipated and the surgeons were advised to avoid the use of monopolar cautery and to use only bipolar cautery with short bursts of less than 10 seconds. In case of prolonged bursts lasting more than 10 seconds, the surgeons were promptly informed. Emergency drugs for resuscitation and an external defibrillator were kept ready for the management of intraoperative arrhythmias if required. Requirements for transvenous pacing were readily available. Cardiology backup was ensured in case of any intraoperative complications. Although there is no strong recommendation for prophylactic platelet transfusion for patients on dual antiplatelet therapy, a clinical decision was made to transfuse platelets in this patient due to the high risk and emergency nature of the surgery to ensure normal coagulation and to minimize blood loss [6]. Noradrenaline infusion was required to maintain the blood pressure of the patient within 20% of the preoperative baseline. The surgery lasted for five hours. Intraoperative blood loss was 1200 ml which was managed with the transfusion of blood and blood products such as fresh frozen plasma and platelets. There were no intraoperative arrhythmias and the cardiac status of the patient remained stable throughout the surgery. Postoperatively, the patient was shifted to a neurosurgical intensive care unit and mechanical ventilation was continued because of poor preoperative GCS. The patient was planned for elective tracheostomy due to prolonged weaning from mechanical ventilation.

## Discussion

This case report highlights the perioperative management of a neurosurgical patient with uncontrolled hypertension and a pacemaker in situ. Perioperative management of patients with pacemakers poses unique challenges. A thorough history and assessment with proper interrogation of the device might not always be possible in case of emergency procedures. A quick idea about the device can be gathered from the device information card which contains details regarding the date of device insertion, model number, indication, and the current settings [7]. This was the case in this patient as a detailed interrogation of the device was not possible due to the deteriorating neurological condition which required prompt surgical intervention. Before elective surgery, the pacemaker is usually set in the asynchronous mode to prevent complications due to electromagnetic interference, especially in supraumbilical surgeries where the surgical field can be near the pacemaker [7]. Electromagnetic interference during the use of cautery can trigger the anti-tachyarrhythmia function and lead to bradycardia or even asystole in a patient who is pacemaker-dependent. In a previous case report, electromagnetic interference-induced asystole was reported in a 74-year-old male patient undergoing spine surgery with DDDR (dual-chamber paced, dual-chamber sensed, dual response to sensing, and rate modulated) pacemaker in-situ [8]. Continuous ECG monitoring plays an important role in such cases for the detection and treatment of intraoperative arrhythmias. Adequate preparations and precautions are required when managing such patients. Ensuring the availability of external defibrillators and the equipment for transvenous pacing can be life-saving in patients in whom pacemaker malfunction is anticipated in the intraoperative period.

Patients on dual antiplatelet drug therapy can have an increased risk of intracranial hemorrhagic complications [9]. The increasing size of hematoma can lead to an acute rise in intracranial pressure and rapid deterioration of consciousness. The decision to reverse the anticoagulant functions should be weighed against the risk of thrombosis in patients with coronary artery disease.

Increased intracranial pressure can result in circulatory changes with a triad of hypertension, bradycardia, and altered respiration, commonly known as the "Cushing Triad." In a patient with uncontrolled hypertension, this can lead to further elevations in blood pressure. With a rapid decrease in the intracranial pressure following decompressive craniectomy, it can lead to a decrease in mean arterial blood pressure levels as well which can be detrimental in patients with hypertension in whom the cerebral autoregulatory set points would be set at a higher level than in normotensive patients [10]. Hence rapid hemodynamic fluctuations should be managed with infusions of vasopressors such as noradrenaline to maintain the mean arterial blood pressure within 20% of the patient’s baseline blood pressure. In addition to this, there can be increased blood loss during craniectomy which can lead to further hemodynamic instability. Increased blood loss can be detrimental in patients with preexisting cardiac ischemic changes and can lead to further deterioration of cardiac functions. Hence intraoperative blood loss should be vigilantly assessed and replaced with adequate blood and blood products. Maintaining intraoperative hemodynamic stability and preventing rapid fluctuations in intracranial pressure and cerebral perfusion pressure can lead to better perioperative outcomes in cardiac patients undergoing neurosurgical procedures.

## Conclusions

Management of a cardiac patient with a pacemaker for an emergency neurosurgical procedure can be a challenging task for the anesthetist. With limited time availability for preoperative evaluation, a quick history and brief examination can provide valuable information that can be pivotal in perioperative patient management. In a patient with a pacemaker in situ, avoiding the usage of monopolar cautery and being prepared for transvenous pacing and external defibrillation are important in preventing adverse events due to pacemaker malfunction. This case report highlights the successful management of a hypertensive patient with coronary artery disease and a permanent pacemaker taken up for emergency neurosurgery.
